# Supplementary material for: Five-year survival rate and prognostic factors in women with breast cancer treated at a reference hospital in the Brazilian Amazon
Source: PLoS One. 2022 Nov 17;17(11):e0277194. doi: 10.1371/journal.pone.0277194 (PMC9671322; doi:10.1371/journal.pone.0277194)
Supplement: Attachment — Submitted filename: Response to Reviewers.docx [file pone.0277194.s002.docx]

Dear Team of Editor, Plos one

We present for your evaluation our review of manuscript number [PONE-D-21-12167].

We are very grateful because this important scientific journal has given us the chance to evaluate our research for possible publication.

We emphasize that we try to meet all the requests of the reviewers and we hope to have really clarified the points questioned.

Regards.

Soany Cruz

**Reviewer: 1**

Reviewer #1: The paper estimates absolute and relative 5-year survival following breast cancer diagnosis in a hospital in the Brazilian Amazon. Only 65% of found cases were included in the analysis. A major concern of the findings is the potential for selection bias differential by patient prognosis.

**Major Comments:**

1) The authors included 65% (1,430) of the 2,185 cases found in the analysis. This selection needs to be better described.
a) Are these exclusions of records with incomplete information, as per page 3, line 80-81? If so please, provide a breakdown of the numbers excluded because of incomplete mortality ascertainment versus missing covariate information?
b) Please demonstrate that the population excluded is not different from those included in a table 1 comparing characteristics of the two populations (include a missing category) with the appropriate statistical test (chi-square test for categorical variables, Mann-Whitney U test for continuous variables).

**Our response:**

We inform in a new table 1 of the requested information.

We compared both populations as requested, and it appeared in table 1.

**Minor comments:**

Tables require some double-checking. Comma instead of decimal in the first row of table 1; global survival inconsistently including/not including % in tables 1 and 2; and the decimal places seem to be in the wrong places in the RR column of table 3.

**Our answer:**

The authors reviewed all the data in the tables as requested.

**Reviewer: 2**

The main limitation with this study is that it is hospital-based rather than population-based, nor does there appear to be any population-based data to compare cases in this study with the number of cases in the region. The conclusions of the study therefore are only applicable to women able to access hospital care. Was data on hormone receptor status available? If it is available it would have been an interesting variable to also look at. In model 3 there are 0 cases (seen in Table 2) in some of the cells for tumour size and carcinoma without infiltration. This can make the model unstable. Was any analysis done of correlation between variables? I suspect that some of the variables may be highly correlated and therefore may be able to be excluded from the model.

**Our answer:**

There were no records on hormonal receptor in the medical records, perhaps because the clinical laboratory of the hospital is not able to do this type of analysis.

About the tumor size in table 2, we noticed that there were no deaths with small tumors, perhaps because it was an early stage of the disease so that it could be more likely to increase survival, but this variable was not important in the adjustment in regression analysis (table 3).
