# Supplementary material for: Five-year survival rate and prognostic factors in women with breast cancer treated at a reference hospital in the Brazilian Amazon
Source: PLoS One. 2022 Nov 17;17(11):e0277194. doi: 10.1371/journal.pone.0277194 (PMC9671322; doi:10.1371/journal.pone.0277194)
Supplement: Attachment — Submitted filename: Response to Reviewers.docx [file pone.0277194.s003.docx]

Dear Team of Editor, Plos one

We present for your evaluation our review of manuscript number [PONE-D-21-12167].

We are very grateful because this important scientific journal has given us the chance to evaluate our research for possible publication.

We emphasize that we try to meet all the requests of the reviewers and we hope to have really clarified the points questioned.

Regards.

Soany Cruz

**Reviewer: 1**

Reviewer #1: Cruz S et al. have reported ' Five-year survival rate and prognostic factors in women with breast cancer treated at a reference hospital in the Brazilian Amazon'. It is good that the authors try to report an important public health issue in a more neglected area in South America, but there are some weak points in the study that makes it suboptimal:

1. if the idea of authors is to provide an awareness about the status of diagnosis and treatment of BC and yo show their concern about delays they need to explain the currently existing policies and screening programs in the region. Given the specific localization, some information about the culture and the behavior of patients in the region is useful to help to accept the conclusion about the needs for screening and early diagnosis programs.

**Our response:** We add the requested information.

2. There is very poor information about nature of BC tumors in the region in the provided data. There is a general classification of 'infiltrating ductal' versus 'others' with a strange line of 'carcinoma with no infiltration'. What this statement exactly mean? Are the authors talking about carcinoma in situ? Why this needs to be mentioned as an independent category given its very low number (expected). Also it does not fit with the clinical stages provided, CIS of breast is Tis. As soon as breast mass of 1mm, it is already T1 (AJCC, 2018).

**Our response:** We used the classification of the Brazilian Society of Clinical Oncology, which classifies the histology of breast cancer between carcinoma in situ and infiltrating carcinoma.

**3.** Infiltrating ductal carcinoma is a big category that covers multiple molecular subtypes that are diagnosed based on IHC and the type of treatment is based on that categorization. Is that mean that in Belem there s no information at all about molecular subtypes or at least the status of HR?

**Our response:** Our research used only data from medical records. However, many of these records were old and did not contain this type of information. Furthermore, the hospital does not carry out this type of analysis, being carried out in external laboratories. This analysis is often not attached to the medical record, making it difficult to investigate this topic.

**4.** Tumor size >2 cm is not really a good indicator of 'T' element in TNM staging, it covers a range of T2 to T4. If such data is not available, then how data on lymph node and metastasis (the combination to define stage of BC) is reliable?

**Our response:** We have the data available, however we chose to classify the tumor size between smaller and larger than 2cm to establish the tumor at an early or advanced stage in the statistical analysis.

**5.** It is not clear how the % are calculated: as only one example: The authors report the final sample size (after exclusions) =1430, Table one reports 661 with LN involvement that makes it 46.2%, how the authors end up to 58.8% ? Additionally, how 137 (58.5%) missing data is calculated?

**Our response:** Some medical records contained information about one variable and not about another. So, the analysis took into account the variable that is contained in the medical record. So, in some cases, the proportions don't match. The missing data corresponds to numbers excluded because of incomplete mortality ascertainment versus missing covariate information

**6.** Can authors give a short definition about how they categorize the patients to white or non-white and what criteria do they use?

**Our response:** According to Brazilian law, the identification of race or color must respect the user's self-declaration criteria.
