# Supplementary material for: Five-year survival rate and prognostic factors in women with breast cancer treated at a reference hospital in the Brazilian Amazon
Source: PLoS One. 2022 Nov 17;17(11):e0277194. doi: 10.1371/journal.pone.0277194 (PMC9671322; doi:10.1371/journal.pone.0277194)
Supplement: Attachment — Submitted filename: Response to Reviewers.docx [file pone.0277194.s005.docx]

Dear Team of Editor, Plos one

We present for your evaluation our review of manuscript number [PONE-D-21-12167].

We are very grateful because this important scientific journal has given us the chance to evaluate our research for possible publication.

We emphasize that we try to meet all the requests of the reviewers and we hope to have really clarified the points questioned.

Regards.

Soany Cruz

**Reviewer #3:** Thanks for your replies. While your reply to my first review question regarding the current status of BC in the region and a summary of the cultural points that might affect the prognosis of this cancer is 'this data is added', I could not find any addition in the revised version with tracking pointing this question. If you have addressed this question, please be sure if it is done through tracking facility to be found.

**Our response:** We add and track the requested information.

**Reviewer #4:** Thank you very much. This is an interesting investigation on the breast cancer and the authors have used a survival analysis methodology. The manuscript is generally well structured. However, it has some shortcomings in regards to some data analyses and narrations. Below I have provided remarks on the analysis as it is often vague and long-winded.

• The test used is Kaplan-Meier statistical method and Cox regression analysis. However, no information is provided about the assumption tests.

**Our response:** We add the requested information.

• Why mean survival time? , why not median survival time? Given these shortcomings the manuscript requires major revisions.

**Our response:** For this case, the estimated survival probability must never have reached 50%, that is, the survival step function does not cross the line y=.5. The Kaplan-Meier estimate, especially since it is a non-parametric method, makes no inference about survival times (i.e., the shape of the survival function) beyond the range of times found in the data. Mean survival time, on the other hand, is a statement about the observed times. It shouldn't be taken to mean the length of time a subject can be expected to survive.

**Reviewer #5:** This article entitled “Five-year survival rate and prognostic factors in women with breast cancer treated at a reference hospital in the Brazilian Amazon” aimed to analyze the five-year survival rate and prognostic factors in Northern Brazilian women from 2007-2013. A total of 1,430 cases were analyzed in this study and in multivariable analysis, referral from public healthcare system, advanced clinical stage, lymph node involvement, and metastasis, were associated with lower survival rates. Overall, the study is important in highlighting this public health issue/disparity in this region of Brazil. While overall the authors did a nice job, there are several areas in the study that could be addressed for clarity.

**Abstract/Introduction**

1. The authors state in both the abstract and introduction that BC is the leading cause of death among women globally. I would suggest to clarify that to “leading cause of cancer death among women globally”.

**Our response:** We correct it.

2. There are font/size discrepancies in the introduction (lines 54-56).

**Our response:** We correct it.

3. Is there a citation for (line 54-56)- early detection of breast cancer in Brazil?

**Our response:** There is no citation, it is a point of reflection raised by the authors

4. What constitutes difficulties in accessing healthcare? Lack of doctors/specialists? Distance to office? Healthcare (public versus private)? Combination of all the above?

**Our response:** We add the information required.

**Methods**

1. Are there any inclusion/exclusion criteria for selection based on clinical factors (other than breast cancer as the primary tumor)?

**Our response:** The authors chose only this criterion because they aimed to analyze as many variables as possible.

2. Could be beneficial for clarity to include citation from Hosmer and Lemeshow to describe the univariate variable selection criteria (p<0.2). (Hosmer DW, Lemeshow S, Sturdivant RX. Applied logistic regression. New York: John Wiley & Sons, Incorporated, 2013.)

**Our response:** To build the final regression model and establish the independent variables that influenced survival time, we used the method to include the most important predictors from the p-value (0.2) observed in the univariate analysis. We included the citation.

3. How are deaths defined for the survival analysis? Was it all-cause mortality? Breast cancer related deaths adjudicated by a physician?

**Our response:** Deaths recorded in the medical records of the breast cancer service were considered. The authors did not distinguish the type of death so as not to incur errors.

4. It may be beneficial to include a section about the variables and how some variables were defined (e.g. why 2 cm tumor size? Is that a proxy to early/late stage clinical stage?)

**Our response:** The 2cm criterion is established by the staging currently used as a limit. We chose to classify the tumor size between smaller and larger than 2cm to establish the tumor at an early or advanced stage in the statistical analysis.

5. For histological type, why focus on just infiltrating ductal carcinoma and carcinoma in situ? What comprises “others”? Is this due to the detail in the medical records? Any thought about stratifying analysis by histological type for sensitivity analyses?

**Our response:** We chose this division because it is very precise in terms of the location of the tumor in a single layer of tissue or when the tumor already presents an invasion behavior from other locations (even if they are other layers).

Others means other types of cancer than breast carcinomas, we chose not to stratify this variable too much since they represent 14.5% of the total sample.

It did not occur to the authors to carry out this type of analysis, but we will take this as a suggestion for future research.

6. It is not stated in the paper if the proportional hazards assumptions were violated. Also, were there tests for multicollinearity? It seems that several of the tested variables would be strongly correlated (e.g., tumor size & clinical stage; clinical stage & lymph node involvement & metastasis)- all of which made it to the final model.

**Our response:** The authors analyzed by the kaplan meier curves, since they are in parallel, the proportional risk is satisfied. We verified by the correlation matrix of the cox model coefficients and observed that there was no correlation above 0.47.

**Results**

1. Why the comparison between the missing and included data in Table 1?

**Our response:** The authors wanted to demonstrate the summary of patients who were not involved in the analysis and compare with those who were analyzed.

2. What source is the global % coming from in Table 2?

**Our response:** Means the survival of each variable stratum

3. Argument would benefit from more explanation on the final model (e.g., “lymph node involvement more than doubles the risk of breast cancer in this population (HR= 2.35 [1.38-3.98]; Table 3).”

**Our response:** The authors intended to be more objective and allow for a more fluid reading. We will take this suggestion for future manuscripts.

Discussion

1. There really is no conclusion/summary at the end of the paper

**Our response:** We add the information required.
