# Supplementary material for: Five-year survival rate and prognostic factors in women with breast cancer treated at a reference hospital in the Brazilian Amazon
Source: PLoS One. 2022 Nov 17;17(11):e0277194. doi: 10.1371/journal.pone.0277194 (PMC9671322; doi:10.1371/journal.pone.0277194)
Supplement: Attachment — Submitted filename: Reviewer comment.docx [file pone.0277194.s004.docx]

Title: Five-year survival rate and prognostic factors in women with breast cancer treated at a reference hospital in the Brazilian Amazon

**General comments**

Thank you very much.

This is an interesting investigation on the breast cancer and the authors have used a survival analysis methodology. The manuscript is generally well structured.

However, it has some shortcomings in regards to some data analyses and narrations.

Below I have provided remarks on the analysis as it is often vague and long-winded.

- The test used is Kaplan-Meier statistical method and Cox regression analysis

However, no information is provided about the assumption tests.

- Why mean survival time? , why not median survival time?

Given these shortcomings the manuscript requires major revisions

Abstract:

Breast cancer is the most prevalent malignant neoplasm and the leading cause of death among women globally. In Brazil, survival rates vary according to the region and few studies have been conducted on breast cancer survival in less developed areas, such as the Amazon region.

The aim of this study was to analyze the five-year survival rate and prognostic factors in women treated for breast cancer in the city of Belém in northern Brazil. A retrospective hospital-based cohort study was conducted (2007-2013). Sociodemographic, clinical/tumor, and treatment variables were obtained from the records at the Ophir Loyola Hospital. Survival analysis involved the Kaplan-Meier statistical method and Cox regression analysis was performed. The significance level was 5% (p <0.05). A total of 1,430 cases were analyzed. Mean survival time was 51.71 (± 17.22) months, with an estimated overall survival of 79.4%. In the multivariate analysis, referral from the public health care system, advanced clinical stage, lymph node involvement and metastasis were associated with worse prognosis and lower survival rate. Radiotherapy and hormone therapy were associated with increased survival. These findings can contribute to the development of regional strategies for preventing breast cancer, reducing the incidence and mortality rates and increasing survival time.
